# Preclinical evaluation of [^18^F]FB-A20FMDV2 as a selective marker for measuring α_V_β_6_ integrin occupancy using positron emission tomography in rodent lung

**DOI:** 10.1007/s00259-019-04653-5

**Published:** 2020-01-03

**Authors:** Mayca Onega, Christine A. Parker, Christopher Coello, Gaia Rizzo, Nicholas Keat, Joaquim Ramada-Magalhaes, Sara Moz, Sac-Pham Tang, Christophe Plisson, Lisa Wells, Sharon Ashworth, Robert J. Slack, Giovanni Vitulli, Frederick J. Wilson, Roger Gunn, Pauline T. Lukey, Jan Passchier

**Affiliations:** 1 Imanova Ltd trading as Invicro, Burlington Danes Building, Imperial College London, Hammersmith Hospital, Du Cane Road, London, W12 0NN UK; 2 GlaxoSmithKline, Medicines Research Centre, Gunnels Wood Road, Hertfordshire, SG1 2NY UK

**Keywords:** A20FMDV2, α_v_β_6_, Integrin, PET, Fibrosis, Rodent lung

## Abstract

**Purpose:**

Integrin α_v_β_6_ belongs to the RGD subset of the integrin family, and its expression levels are a prognostic and theranostic factor in some types of cancer and pulmonary fibrosis. This paper describes the GMP radiolabelling of the synthetic 20 amino acid peptide A20FMDV2 (NAVPNLRGDLQVLAQKVART), derived from the foot-and-mouth disease virus, and characterises the use of [^18^F]FB-A20FMDV2 as a high affinity, specific and selective PET radioligand for the quantitation and visualisation of α_v_β_6_ in rodent lung to support human translational studies.

**Methods:**

The synthesis of [^18^F]FB-A20FMDV2 was performed using a fully automated and GMP-compliant process. Sprague-Dawley rats were used to perform homologous (unlabelled FB-A20FMDV2) and heterologous (anti-α_v_β_6_ antibody 8G6) blocking studies. In order to generate a dosimetry estimate, tissue residence times were generated, and associated tissue exposure and effective dose were calculated using the Organ Level Internal Dose Assessment/Exponential Modelling (OLINDA/EXM) software.

**Results:**

[^18^F]FB-A20FMDV2 synthesis was accomplished in 180 min providing ~800 MBq of [^18^F]FB-A20FMDV2 with a molar activity of up to 150 GBq/μmol and high radiochemical purity (> 97%). Following i.v. administration to rats, [^18^F]FB-A20FMDV2 was rapidly metabolised with intact radiotracer representing 5% of the total radioactivity present in rat plasma at 30 min. For the homologous and heterologous block in rats, lung-to-heart SUV ratios at 30–60 min post-administration of [^18^F]FB-A20FMDV2 were reduced by 38.9 ± 6.9% and 56 ± 19.2% for homologous and heterologous block, respectively. Rodent biodistribution and dosimetry calculations using OLINDA/EXM provided a whole body effective dose in humans 33.5 μSv/MBq.

**Conclusion:**

[^18^F]FB-A20FMDV2 represents a specific and selective PET ligand to measure drug-associated αvβ6 integrin occupancy in lung. The effective dose, extrapolated from rodent data, is in line with typical values for compounds labelled with fluorine-18 and combined with the novel fully automated and GMP-compliant synthesis and allows for clinical use in translational studies.

**Electronic supplementary material:**

The online version of this article (10.1007/s00259-019-04653-5) contains supplementary material, which is available to authorized users.

## Introduction

Integrins are a class of receptors that play an important role in cell-cell and cell-matrix interactions in all higher organisms. There are 24 known heterodimeric receptors composed of an alpha subunit and a beta subunit. In a subset of eight integrins, the Arg-Gly-Asp (RGD) sequence was identified as the minimal integrin-binding motif [1]. Integrin α_v_β_6_ belongs to this RGD subset of the integrin family and is expressed principally on epithelial cells [2]. The similarity between the RGD binding regions presents a challenge to finding a ligand that binds with high selectivity and affinity to α_V_β_6_, as most RGD ligands previously described showed residual yet significant affinity to other RGD integrins as well [3, 4].

With the exception of the gastrointestinal tract, expression of α_V_β_6_ is not usually detected in healthy adult tissues by immunohistochemistry; its expression increases to detectable levels on injured epithelial cells. Increased expression of α_V_β_6_ contributes to fibrosis, tumorigenesis and metastasis [5–9]. Kaplan-Meier analysis of integrin α_V_β_6_ mRNA expression in 488 colorectal carcinomas revealed a striking reduction in median survival time of patients with high integrin α_V_β_6_ expression [10]. Similarly, Kaplan-Meier analysis of integrin α_V_β_6_ immunohistochemistry of lung tissue sections from 43 subjects with idiopathic pulmonary fibrosis revealed that the extent of integrin α_V_β_6_ immunostaining was associated with increased mortality [11]. These data suggest that high levels of integrin α_V_β_6_ may identify subjects with progressive malignancy or fibrotic disease [10–12]. However, assessment of integrin α_V_β_6_ expression has to date only been possible through focused and invasive biopsy of diseased tissue.

A non-invasive imaging method to assess integrin α_V_β_6_ expression such as PET/CT would have prognostic applications in clinical practice. In addition, α_V_β_6_ PET/CT could be used during early clinical development to assess target engagement of a new therapeutic inhibitor of α_V_β_6._ In fact, the work presented in this manuscript forms the preclinical basis for a recently completed clinical study: a First Time in Human (FTIH) study of inhaled GSK3008348 (an α_V_β_6_ inhibitor) in Healthy Volunteers and Idiopathic Pulmonary Fibrosis Patients (NCT02612051). In addition, such a non-invasive technique could be used in clinical assessment of disease severity, disease activity and prognosis. Thus, there is an increasing interest in developing a positron emission tomography (PET) ligand for non-invasive imaging of α_V_β_6_ expression for preclinical and clinical applications.

Several α_V_β_6_-targeting peptides have been identified to date, and their properties as ligands for PET and single photon emission computed tomography (SPECT) imaging have been investigated [4, 13–15]. The synthetic 20-amino acid peptide A20FMDV2 (NAVPNLRGDLQVLAQKVART) is derived from foot-and-mouth disease virus (FMDV) and has been reported as a selective inhibitor of α_V_β_6_ in a pancreatic cancer xenograft model [16]. A20FMDV2 shows high binding affinity and good selectivity towards the α_v_β_6_ integrin compared with the other members of the RGD integrin family, namely, α_v_β_3_, α_v_β_5_, α_5_β_1_ and α_IIb_β_3_ [16, 17]. A20FMDV2 was initially labelled with fluorine-18, using 4-[^18^F]fluorobenzoic acid ([^18^F]FBA) and developed as a preclinical PET tracer for in vivo cancer imaging [16]. High-specific binding to α_V_β_6_ was demonstrated in *in vitro* cell binding assays, and [^18^F]FB-A20FMDV2 (otherwise known as [^18^F]IMAFIB and [^18^F]GSK2634673) was shown to selectively image α_V_β_6_-positive tumours *in vivo* in mice-bearing human melanoma xenografts [16]. Indium-111-labelled A20FMDV2 peptide is able to detect increased levels of α_v_β_6_ integrin in the lungs of mice in the bleomycin-induced model of pulmonary fibrosis [18, 19]. This has been confirmed independently using radioligand binding assays where [^3^H]A20FMDV2 was shown to bind to α_V_β_6_ with high affinity (K_D_: 0.22 nmol/l) and selectivity (at least 85-fold) for α_V_β_6_ over the other members of the RGD integrin family [20].

More recently, attempts have been made to improve the imaging properties of [^18^F]FB-A20FMDV2 as an α_V_β_6_ ligand by using different prosthetic groups and chelators for radiolabelling and by introducing spacers [17, 21–27]. Furthermore, A20FMDV2 has been labelled with other PET and SPECT nuclides, and the effects of those on pharmacokinetics, metabolism and tumour uptake have also been investigated [17, 18, 21–27]. While moderate improvements in pharmacokinetics were observed, [^18^F]FB-A20FMDV2 remains one of the most potent and selective α_V_β_6_ ligands reported to date [4]. The availability of a specific and selective PET ligand to delineate α_V_β_6_ integrin in humans *in vivo* would allow exploration of the role of this integrin receptor in disease and provide a means to support drug development activities aimed at targeting this integrin. To date, animal models of disease have involved the use of bleomycin to induce lung fibrosis. This model leads to significant weight loss in the animals and highly variable levels of fibrosis and requires significant resource investment to ensure optimal results. Initial evidence through our own efforts suggested that, despite the low tissue density and high blood compartment in the lung, sufficient α_V_β_6_ integrin may be expressed in healthy animals to allow determination of drug-associated occupancy. The ability to do so without the need for the bleomycin model would significantly improve the applicability of the technology and provide further confidence for clinical translation.

Here we report the translational preclinical characterisation and GMP-compliant manufacture of [^18^F]FB-A20FMDV2 in support of future clinical studies.

## Materials and methods

Details on materials including the precursor A20FMDV2 and the reference standard FB-A20FMDV2 (alternative identifiers: IMAFIB, GSK2634673) can be found in the Supplementary Information.

All experiments were carried out in accordance with the Animals (Scientific Procedures) Act 1986, in line with EU directive 2010/63/EU and approved by the Animal Welfare and Ethical Review Board of Imperial College London. Details can be found in the Supplementary Information.

### Automated GMP-compliant synthesis, QC and radiometabolite analysis of [^18^F]FB-A20FMDV2

The automated GMP-compliant radiosynthesis of [^18^F]FB-A20FMDV2 was performed on a Modular-Lab™ system (Eckert and Ziegler, Germany). Details on the radiosynthesis procedure, quality control and radiometabolite analysis methods can be found in the Supplementary Information.

### In vitro selectivity of A20FMDV2

A20FMDV2 competition binding studies against the RGD integrins were conducted using radioligand binding (α_v_β_1_, α_v_β_3_, α_v_β_5_, α_v_β_6_, α_v_β_8_, α_5_β_1_ and α_8_β_1_) or platelet aggregation (α_IIb_β_3_) assays, as previously described [28, 29]. Briefly, full competition binding curves were generated by either incubating a small molecule RGD-mimetic-tritiated radioligand with soluble integrin protein or human whole blood-derived platelets (acquisition of venous blood samples was approved by the Hertfordshire Research Ethics Committee and all donors gave informed consent prior to donation.) and fibrinogen for α_IIb_β_3_. Data are the mean ± SEM of four individual experiments/donors. See Supplementary Information for additional assay details.

### Preclinical rodent studies

Male Sprague-Dawley rats (supplier Charles River, UK) were acclimated for a minimum of 3 days prior to commencing studies. Homologous and heterologous competition studies were carried out unblinded. No adverse effects were observed from the administration of either the homologous or heterologous agents.

### *In vivo* homologous and heterologous blocking studies

Rats were anaesthetised using isoflurane anaesthesia (2–2.5% isoflurane, 1 L/min oxygen) and a cannula surgically placed into both the lateral tail vein and the tail artery of each animal for radioactive dose administration and blood sampling, respectively. Each subject was individually placed within the PET-CT scanner (Inveon DPET/MM, Siemens AG, Erlangen, Germany), and two PET scans were acquired (scan details included in Supplementary Information). Each subject underwent a 60-min dynamic PET acquisition (baseline scan) following intravenous (i.v.) bolus administration of [^18^F]FB-A20FMDV2. For the homologous blocking (*n* = 3 animals), immediately following the end of the first scan, unlabelled FB-A20FMDV2 was administered followed 5 min later by a repeat dynamic scan (post-dose scan) with [^18^F]FB-A20FMDV2. For the heterologous block study (*n* = 6 animals), animals were allowed to recover after the first scan and were scanned again on a separate day following dosing with 8G6, a specific antibody against α_V_β_6_. Details for the procedures can be found in the Supplementary Information.

### Ex vivo [^18^F]FB-A20FMDV2 homologous competition study

Twelve male rats (380–431 g) were anaesthetised and maintained under terminal isoflurane anaesthesia (2–2.5% isoflurane, 1 L/min oxygen). Control rats (*n* = 6) were injected with [^18^F]FB-A20FMDV2 only. Treated rats (*n* = 6) were injected with unlabelled FB-A20FMDV2 (2 mg/kg in 0.9% saline) 5 min prior to injection of [^18^F]FB-A20FMDV2. All radioactive doses were injected by direct tail vein injection (4–7 MBq). Rats were euthanised by exsanguination 30 min after tracer injection and the following samples collected, weighed and measured for radioactivity using a gamma counter: blood, plasma, heart, lung, liver, spleen, stomach wall, kidney cortex, kidney medulla, red bone marrow, bone and muscle (scapularis and bicep).

Blood, plasma and tissue radioactivity concentrations were decay-corrected to the time of [^18^F]FB-A20FMDV2 injection and standardised uptake values (SUV) calculated. The SUV in the tissue was normalised to the SUV in the plasma to obtain the tissue to plasma ratio (SUVR_plasma_). Group variation is described as mean ± SD. Groups were compared using an unpaired one-tailed t-test. The significance level was a *P* value of 0.05 or less.

### Rodent radiodosimetry

Twelve rats received a direct tail vein injection of 3–9 MBq of [^18^F]FB-A20FMDV2. Rats (*n* = 3 per time point) were exsanguinated under terminal isoflurane anaesthesia at 5, 15, 30 and 60 min following injection. Tissues of interest were removed, rinsed in chilled saline and counted for levels of radioactivity using a multiwell gamma counter (1470 Wizard, Perkin Elmer, Waltham, MA). Tissue residence times were generated and tissue exposure and effective dose calculated using Organ Level Internal Dose Assessment/Exponential Modelling (OLINDA/EXM) [30]. Further details can be found in Supplementary Information.

## Results

### In vitro selectivity of A20FMDV2

**Fig. 1:**
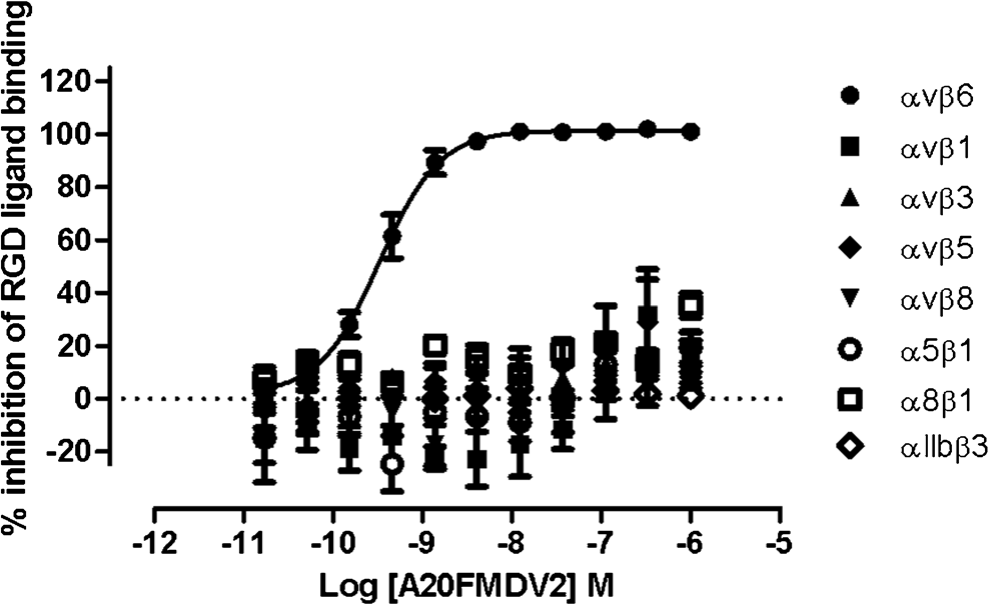
The selectivity profile of A20FMDV2 for the RGD integrins (pKi = 9.82 ± 0.04, *n* = 4; mean ± SEM)

A20FMDV2 was shown to bind with high affinity and selectivity to the α_v_β_6_ integrin (Fig. 1) with < 50% inhibition of binding observed against all the remaining RGD integrins when tested up to a concentration of 1 μM.

### Automated GMP-compliant synthesis, QC and radiometabolite analysis of [^18^F]FB-A20FMDV2

**Scheme 1:**
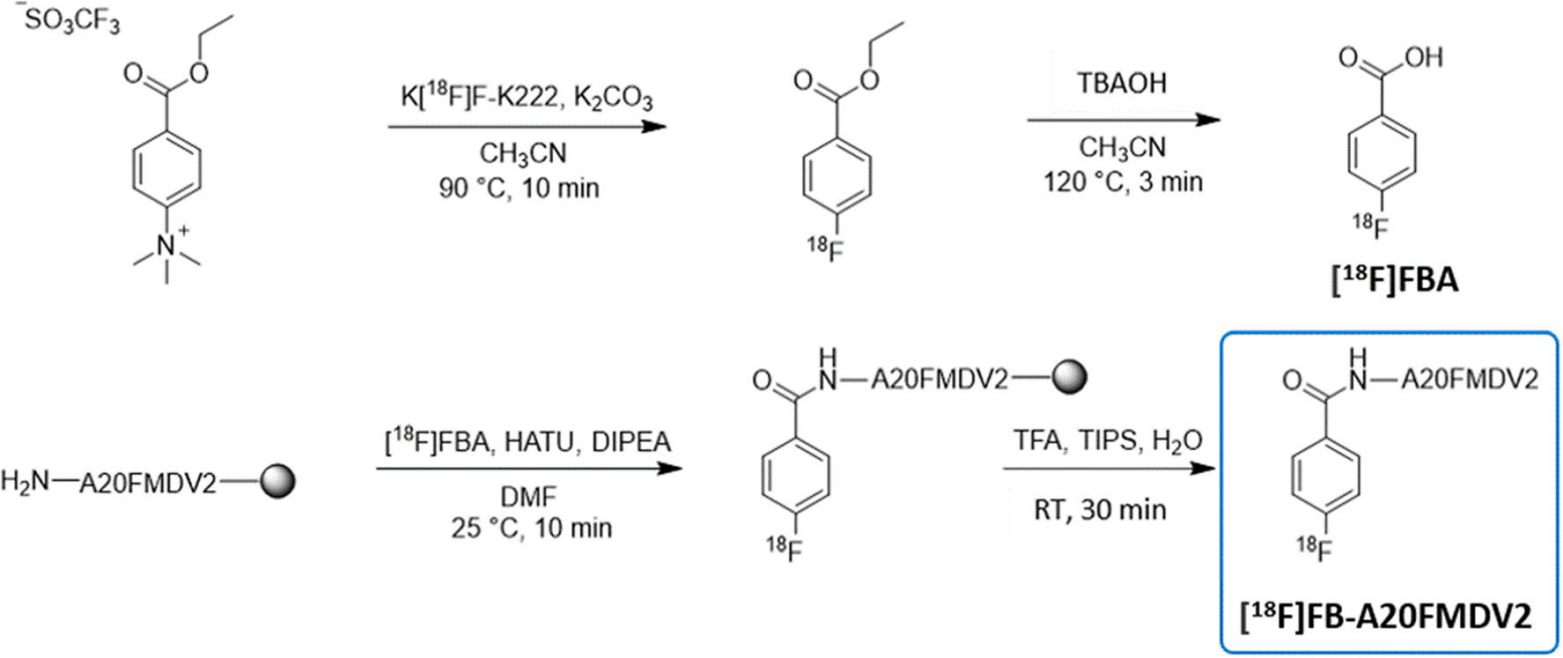
Multistep radiosynthesis process for the manufacture of [^**18**^F]FB-A20FMDV2

Labelling of [^18^F]FB-A20FMDV2 with fluorine-18 was achieved through a multistep-automated process via conjugation of the resin bound precursor (peptide A20FMDV2) to the prosthetic group 4-[^18^F]fluorobenzoic acid ([^18^F]FBA), followed by acidic cleavage from the resin and subsequent purification by semi-preparative HPLC and reformulation (Scheme 1).

Typically, the total synthesis procedure was accomplished in 180 min from end of bombardment (EOB). Up to 800 MBq of [^18^F]FB-A20FMDV2 was synthesised with a molar activity (A_m_) of up to 150 GBq/μmol and with high radiochemical purity (> 97%). The manufacture of [^18^F]FB-A20FMDV2 was validated by three consecutive batches of [^18^F]FB-A20FMDV2 that successfully passed the required specifications (tracer specifications and a summary of the results obtained are shown in the Supplementary Information).

The final product was tested using validated procedures in accordance with good manufacturing practices (GMP) for the quality control tests described in the Supplementary Information. QC tests were performed in agreement with International Conference on Harmonisation and European Pharmacopoeia guidelines [31–35]. Identity and purity (chemical and radiochemical) of [^18^F]FB-A20FMDV2 doses were determined by HPLC analysis, and example sets of QC HPLC chromatograms are depicted in the Supplementary Information.

**Fig. 2:**
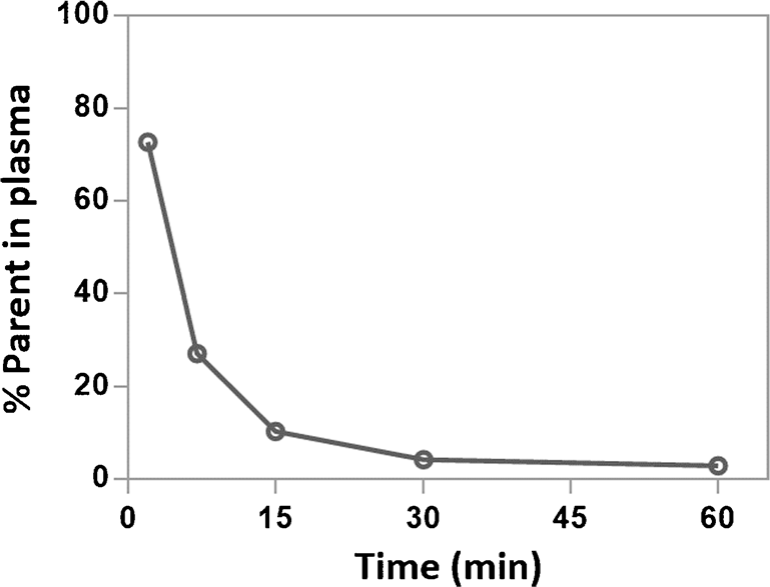
Representative parent fraction of [^18^F]FB-A20FMDV2 in rat plasma

The metabolism of [^18^F]FB-A20FMDV2 was investigated by HPLC as described in the Supplementary Information. In rodents, [^18^F]FB-A20FMDV2 was rapidly metabolised following i.v. administration leading to more polar fragments with approximately 5% of the total radioactivity present in rat plasma at 30 min accounting for intact radiotracer (Fig. 2).

### Preclinical rodent studies

#### *In vivo* homologous blocking study

The molar activity of the radiotracer for each synthesis and injected activity for each scan are reported together with the corresponding mass of FB-A20FMDV2 in the Supplementary Information. The radiochemical purity determined by radio-HPLC was always measured as > 99% since no other radioactive entity could be detected.

**Fig. 3:**
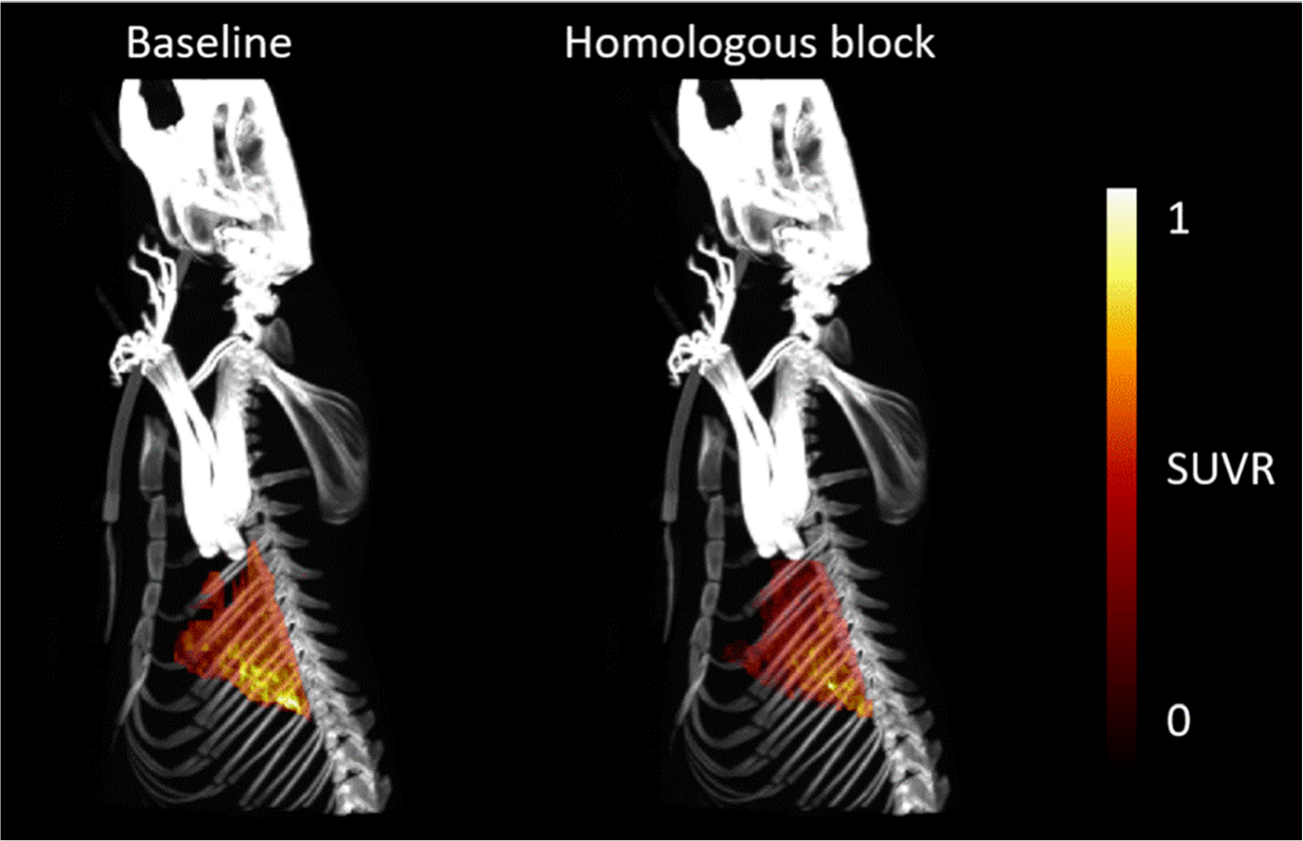
MIP images of co-registered CT and lung-to-heart SUVR PET (30 to 60 min) limited to the lung uptake for subject #2. Left: baseline scan. Right: post-dose scan after administration of FB-A20FMDV2 (2 mg/kg)

Distribution of [^18^F]FB-A20FMDV2 in the rat under both baseline and FB-A20FMDV2 (2 mg/kg) blocking conditions is shown in Fig. 3 as maximum intensity projection (MIP) image with co-registered CT. Following i.v. administration of [^18^F]FB-A20FMDV2, there was a heterogenous distribution of radioactivity under both baseline and homologous block conditions with the highest concentration localised in the liver (on average, SUV ranged from 0.245 in lung to 1.959 in liver under baseline conditions and 0.203 in lung to 1.835 in liver after homologous block).

Baseline and post-injection of homologous blocker (FB-A20FMDV2, 2 mg/kg) lung-to-heart SUVR at 30–60 min after injection of [^18^F]FB-A20FMDV2 are given in Table 1, together with ΔSUVR_30–60_.

**Table 1:** Lung-to-heart SUVR_30–60_: pre and post administration of FB-A20FMDV2

Homologous block study			
Subject #	Lung-to-heart SUVR_30–60_		∆SUVR_30–60_ (%)
	Baseline	Post-dose	
1	1.30	0.70	− 46.3
2	1.07	0.66	− 37.9
3	0.75	0.51	− 32.6
Mean ± SD	1.04 ± 0.03	0.62 ± 0.10	− 38.9 ± 6.9*

*Paired one tailed t-test *P* = 0.0285

#### *In vivo* heterologous block study

The molar activity of the radiotracer for each synthesis and the injected activity for each scan are reported together with the corresponding mass of FB-A20FMDV2 administered in the Supplementary Information.

**Fig. 4:**
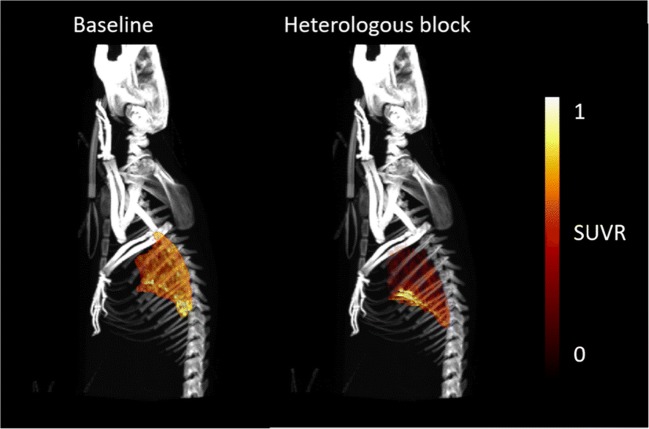
MIP images of co-registered CT and lung-to-heart SUVR PET (30 to 60 min) limited to the lung uptake for subject #6. Left: baseline scan. Right: post dose scan (24 h after administration of 8G6 antibody (5 mg/kg)

MIP images of co-registered CT and lung-to-heart SUVR PET (30 to 60 min), showing only the lung distribution of [^18^F]FB-A20FMDV2 at baseline and following administration of 8G6 antibody, are depicted in Fig. 4. After heterologous block administration, the highest concentration was localised in the liver (on average, SUV ranged from 0.205 in lung to 1.252 in liver under baseline conditions and 0.163 in lung to 1.530 in liver after heterologous block).

Baseline and post-injection of 8G6 SUVR values are given in Table 2, together with ΔSUVR_30–60_. Uptake of [^18^F]FB-A20FMDV2 was significantly reduced in lung (SUVR_30–60min_) post-treatment with anti-α_v_β_6_ antibody 8G6 (5 mg/kg).

**Table 2:** Lung-to-heart SUVR_30–60_: pre- and post-administration of anti-α_v_β_6_ (8G6)

Heterologous block study				
Subject #	Lung-to-heart SUVR_30–60_		∆SUVR_30–60_ (%)	Antibody Conc. (ng/mL) in blood/water (50:50 *v*/v)
	Baseline	Post-dose		
1	1.47	0.91	− 38.0	15,588.6
2	1.02	1.00	− 1.3	< LOQ
3	1.04	0.48	− 53.8	15,781.8
4	1.89	1.03	− 45.2	< LOQ
5	1.06	1.28	20.8	< LOQ
6	1.12	0.27	− 76.3	16,588.3
Mean ± SD	1.27 ± 0.35	0.83 ± 0.38	− 32.3 ± 35.7*	

LOQ: lower than the limit of quantification of 300 ng/mL

*Paired one tailed t-test *P* = 0.0301

The antibody assay results demonstrated presence of antibody in three subjects. Unexpectedly, no quantifiable level of antibody was detected in three other subjects.

#### Rodent dosimetry

Rodent biodistribution was utilised to estimate human radiation exposure using OLINDA/EXM [30]. The highest activity concentration was observed in urine, followed by the small intestine (wall and content), the kidney and liver.

Dosimetry calculations provided the individual organ doses and the whole body effective dose. The organ-absorbed doses estimated using OLINDA/EXM software are summarised in the Supplementary Information. Data revealed the organ with the highest absorbed dose, and contribution to the effective dose was the bladder. The resultant effective dose in humans was estimated to be 33.5 μSv/MBq.

### *Ex vivo* [^18^F]FB-A20FMDV2 homologous block study

**Fig. 5:**
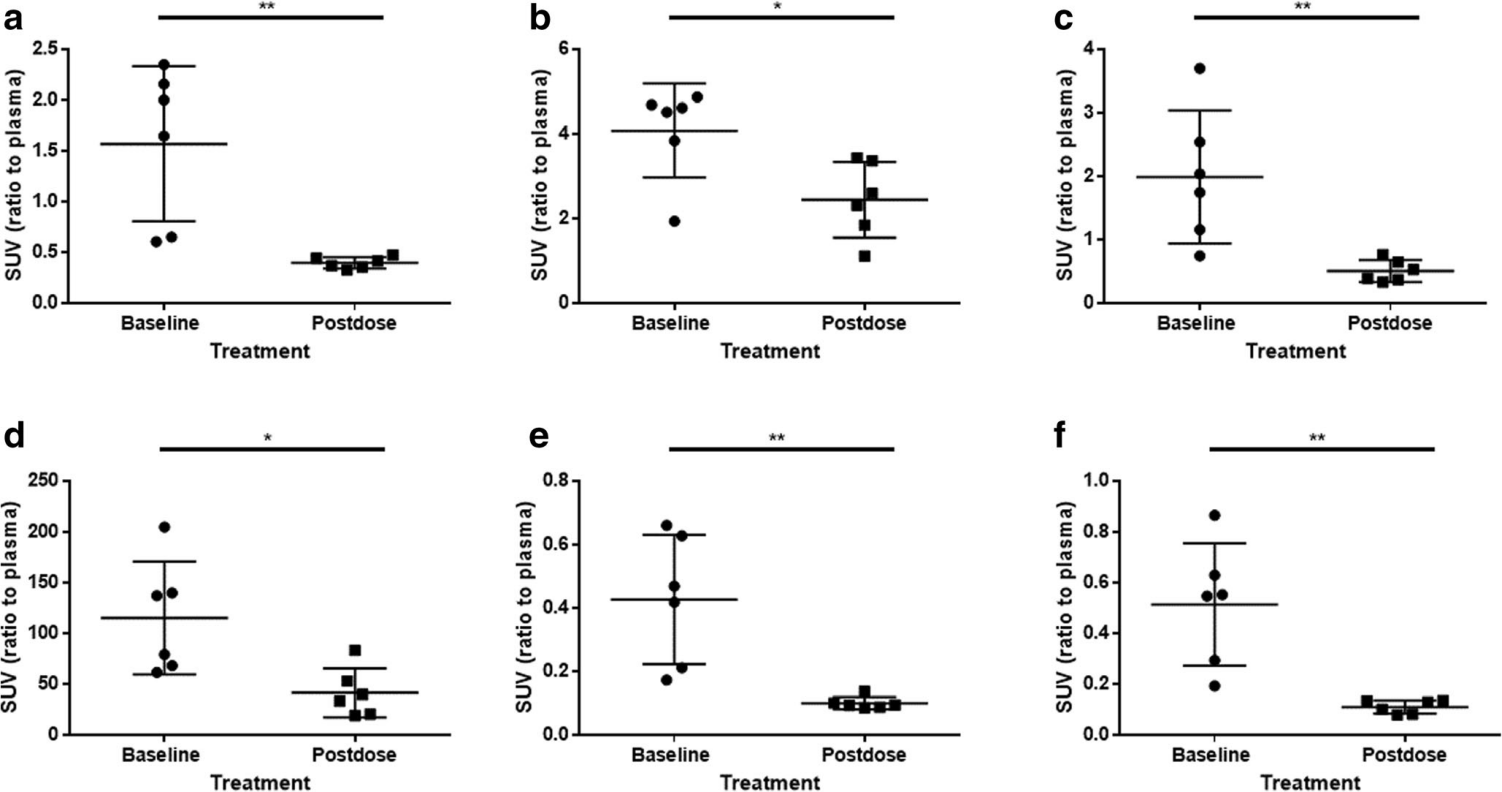
*Ex vivo* measurement of the uptake of [^18^F]FB-A20FMDV2 (mean ± SD, *n* = 6) pre- and post-unlabelled FB-A20FMDV2 (2 mg/kg i.v.). (**a**) lung, (**b**) liver, (**c**) stomach wall, (**d**) kidney medulla, (**e**) scapularis muscle and (**f**) bicep muscle. Groups compared using an unpaired one-tailed t-test. * = *P* < 0.05, ** = *P* < 0.005

Uptake of [^18^F]FB-A20FMDV2 was significantly reduced in lung, liver, stomach wall, kidney medulla and muscle following pre-treatment with unlabelled FB-A20FMDV2 (SUVR baseline vs post-dose (mean ± SD): lung, 1.56 ± 0.76 vs 0.40 ± 0.06; liver, 4.08 ± 1.11 vs 2.45 ± 0.90; stomach wall 1.99 ± 1.05 vs 0.51 ± 0.17; kidney medulla 115.1 ± 55.7 vs 41.6 ± 24.0; and muscle, 0.43 ± 0.20 vs 0.10 ± 0.02 (scapularis) and 0.51 ± 0.24 vs 0.11 ± 0.03 (bicep). Results are depicted in Fig. 5.

## Discussion

The peptide NAVPNLRGDLQVLAQKVART (A20FMDV2), derived from the foot-and-mouth disease virus, has been identified as a potent and selective binder of α_v_β_6_ [16, 20]. This has been confirmed in the current manuscript by evaluating affinity, selectivity and specificity of radiolabelled A20FMDV2 by *in vitro* competition binding assays, rodent *in vivo* heterologous block and both *in vivo* and *ex vivo* homologous block rodent studies. Furthermore, this work further demonstrated the high selectivity of radiolabelled A20FMDV2 for the α_v_β_6_ integrin over the seven other RGD integrins.

Additionally, we have adapted the previously reported manual synthesis of [^18^F]FB-A20FMDV2 [16] to enable the successful implementation of an automated and EU GMP-compliant synthesis. This challenging radiosynthesis, to our knowledge, represents the first example of fully automated process for solid-phase [^18^F] radiolabelling of a peptide in a GMP setting.

Several challenges had to be overcome due to the use of a resin-bound precursor. To prevent blockages of the Modular-Lab™ valves and tubing by the rink amide resin and facilitate its separation from the crude reaction mixture, a fritted cartridge was mounted directly onto a three-way solenoid valve. This cartridge/valve assembly was then placed directly over a magnetic stirrer. To allow good reagent penetration and enhance the yield and efficiency of coupling, the resin beads were pre-swelled in a small volume of solvent during the set-up procedure [27, 36].

The use of TFA for resin cleavage and deprotection commonly results in peptides being delivered as trifluoroacetate salts. No permissible daily exposure (PDE) limit exists for TFA within ICH Q3C guidelines [32], and, consequently, there was a need to limit the amount of TFA in the final product. Generally, TFA can be removed by lyophilisation or in vacuo for an extended period. However, these options are not available within the time constraints for PET chemistry. Consequently, TFA was removed by performing anion exchange using a solid phase extraction (SPE) cartridge [37].

The quality control methods of [^18^F]FB-A20FMDV2 were implemented according to EU GMP guidelines, and a method for the determination of residual TFA in the final dose of [^18^F]FB-A20FMDV2 was implemented to demonstrate its successful removal (< 2 ppm in final product). In order to support translation of [^18^F]FB-A20FMDV2 to the clinic, an HPLC radiometabolite analysis method was also developed to allow generation of the parent arterial input function.

In order to estimate the magnitude and specificity of displaceable component for α_v_β_6_ in vivo in rats, [^18^F]FB-A20FMDV2 scans were performed under baseline conditions and following administration of a pharmacological dose of either FB-A20FMDV2 or the anti-α_v_β_6_ antibody (8G6). All three subjects in the homologous competition study exhibited a decrease in lung SUVR_30–60_ compared to baseline conditions. Similarly, in the heterologous competition study, a decrease in lung SUVR_30–60_ was observed in the three subjects with measurable levels of the 8G6 antibody in blood. A greater decrease in SUVR was observed for subject 6, which may be explained by the fact that the post-dose scan was performed 49 h after i.p. injection of 8G6 compared to the five other subjects which were scanned 24 h after i.p. injection. Subjects 2 and 5 did not have measurable levels of 8G6 antibody in blood and subsequently did not demonstrate the expected large reduction in signal following heterologous block. An interesting anomaly in this dataset is the decrease of ~45% was observed in subject 4 (which did not have quantifiable blood levels of the antibody, 8G6). The reason for this is unclear at this stage, but it is worth noting that subject 4 had a higher than expected SUVR_30–60_ value of 1.89 at baseline. If this measurement were spuriously high, this may partially explain the larger than expected decrease in PET signal for this subject.

The *ex vivo* homologous block study demonstrated a heterogeneous uptake of the [^18^F]FB-A20FMDV2 radioligand throughout the rodent which was reduced in all organs under review following administration of unlabelled FB-A20FMDV2.

Taken as whole, the *in vitro*, *ex vivo* and *in vivo* datasets provide evidence to suggest that [^18^F]FB-A20FMDV2 can specifically and selectively label α_v_β_6_ in healthy rat lung. These data further suggest that [^18^F]FB-A20FMDV2 may be used to demonstrate target engagement even in healthy tissues without the need for upregulation of integrin α_v_β_6 in_ an animal model of disease. This has two significant benefits: it reduces the burden on animals from this severe model (bleomycin induced lung fibrosis), and it avoids the very significant variability that is seen from this model [38].

Rodent dosimetry was conducted to estimate the human radiation exposure of [^18^F]FB-A20FMDV2. Analysis using OLINDA/EXM showed the bladder wall as the organ with the highest absorbed dose. The resulting calculated human effective dose for [^18^F]FB-A20FMDV2 was 33.5 μSv/MBq, which enables repeat scans in patients and healthy volunteers for occupancy and longitudinal studies. This has recently been confirmed in a first-in-human PET dosimetry study performed as part of the clinical translation of [^18^F]FB-A20FMDV2 where the effective dose was determined to be 0.022 mSv/MBq [39]. These findings also demonstrate that initial rodent dosimetry provides a safe estimation of human effective dose to support initial clinical studies.

This work provides the foundation for a series of ongoing clinical applications for the detection of integrin α_v_β_6_ using [^18^F]FB-A20FMDV2 as radioligand in PET/CT studies.

- A Validation and Dosimetry Study of [^18^F]FB-A20FMDV2 PET Ligand has completed (NCT02052297), and part A of the study is published [39].
- In the accompanying manuscript, a completed clinical study is described as quantification of the integrin αvβ6 by [^18^F]FB-A20FMDV2 PET in healthy and fibrotic human lung (PETAL Study). This is parts B and C of study number NCT02052297 and measures avb6 expression in healthy lungs in subjects with fibrotic interstitial lung diseases.
- A First Time in Human (FTIH) study of GSK3008348 (inhibitor of integrin α_v_β_6)_ in Healthy Volunteers and Idiopathic Pulmonary Fibrosis Patients (NCT02612051) has completed and will be published soon. This study used [^18^F]FB-A20FMDV2 to explore target engagement in the lungs of IPF subjects.
- Current clinical studies in liver disease will be reported separately shortly.

## Conclusions

The results reported in this paper demonstrate the utility of [^18^F]FB-A20FMDV2 to specifically and selectively delineate α_v_β_6_ integrins in healthy and diseased lung tissue. Taken together, these results form the basis of ongoing clinical studies using [^18^F]FB-A20FMDV2 to measure α_v_β_6_ integrin levels in fibrotic disease such as idiopathic lung fibrosis and liver fibrosis as well as the determination of occupancy from novel drug candidates. The ability to measure drug-target interaction of novel drug candidates in healthy animals using [^18^F]FB-A20FMDV2 provides a significant improvement over existing approaches using animal models of disease. Therefore, [^18^F]FB-A20FMDV2 may be used as a selective, specific and reversible PET ligand for the α_v_β_6_ integrin and provides an imaging tool that can be utilised in humans to track clinical benefit of emerging therapeutics in debilitating and life-limiting diseases such as lung fibrosis.

## Electronic supplementary material

ESM 1 (DOCX 1596 kb)
