# Supplementary material for: Preclinical evaluation of [18F]FB-A20FMDV2 as a selective marker for measuring αVβ6 integrin occupancy using positron emission tomography in rodent lung
Source: Eur J Nucl Med Mol Imaging. 2020 Jan 3;47(4):958–66. doi: 10.1007/s00259-019-04653-5 (PMC7075836; doi:10.1007/s00259-019-04653-5)
Supplement: Supplementary file 1 — (DOCX 1596 kb) [file 259_2019_4653_MOESM1_ESM.docx]

**SUPPLEMENTARY INFORMATION**

**European Journal of Nuclear Medicine and Molecular Imaging**

**TITLE: Evaluation of [^18^F]FB-A20FMDV2 as a selective marker for α_V_β_6_ integrin using positron emission tomography *in vivo***

Mayca Onega^1^, Christine A. Parker^2^, Christopher Coello^1^, Gaia Rizzo^1^, Nicholas Keat^1^, Joaquim Ramada-Magalhaes^1^, Sara Moz^1^, Sac-Pham Tang^1^, Christophe Plisson^1^, Lisa Wells^1^, Sharon Ashworth^1^, Robert J. Slack^2^, Giovanni Vitulli^2^, Frederick J. Wilson^2^, Roger Gunn ^1^, Pauline T. Lukey*^2^, Jan Passchier*^1^

* Contributed equally to the direction of the work described.

**Author affiliations**

1. Invicro, Burlington Danes Building, Imperial College London, Hammersmith Hospital, Du Cane Road, London, W12 0NN, UK.
2. GlaxoSmithKline, Medicines Research Centre, Gunnels Wood Road, Stevenage, SG1 2NY.

**Corresponding author:**

Dr Jan Passchier

Jan.Passchier@invicro.co.uk

**Materials and Methods**

**Materials**

Kryptofix® (>98%), acetonitrile anhydrous (99.8%), potassium carbonate (Ph. Eur.), trifluoroacetic acid (99%), *N,N*-diisopropylethylamine (DIPEA; 99.5%), triisopropylsilane (TIPS; 99%), sodium acetate (Ph. Eur.), tris(hydroxymethyl)aminomethane (TRIS) and tetrabutylammonium hydroxide (40% in water, w/w) were purchased from Sigma-Aldrich. 1‑[Bis(dimethylamino)methylene]-1H-1,2,3-triazolo[4,5-b]pyridinium 3-oxid hexafluorophosphate (HATU; 98%) was supplied by Applied Biosystems (Warrington, UK). Dimethylformamide (DMF, Hi-Dry®), methanol (Hi-Dry®), acetonitrile (HPLC, UpS™ grade), water (HPLC, UpS™ grade), absolute ethanol (Ph. Eur.) were obtained from Romil (Cambridge, UK).

Water for injection and saline solution (0.9%) were of British Pharmacopeia (BP) grade and were purchased from Fresenius Kabi (Germany & France) and oxygen-18 enriched water (>97%) from Marshall Isotopes Ltd (Tel-Aviv, Israel).

Molecular sieves (4 Å, 80/100) were supplied by Grace (Deerfield, USA). SepPak® cartridges (Accell™ Plus QMA carbonate, Classic C18 and tC18 Plus Light) and Acrodisc syringe filter (PVDF, 25 mm, 0.45 μm) were purchased from Waters (Milford, USA) and fritted cartridges were purchased from Macherey-Nagel. Nalgene™ MF75™ series disposable sterilisation filter units (0.2 μm) and Pall 4908 posidyne sterile filter (0.22 μm) were obtained from Thermo Fisher Scientific and Pall Corporation, respectively.

**Synthesis of precursor and reference standard**

The peptide precursor NAVPNLRGDLQVLAQKVART (AV20FMDV2) on resin (4‑methylbenzhydrylamine hydrochloride, polymer-bound, Rink Amide MBHA), and the FB‑AV20FMDV2 reference standard, 4-fluorobenzamide-NAVPNLRGDLQVLAQKVART-NH2, were purchased from Almac (U.K.). Both materials were manufactured in agreement with GMP requirements and were supplied in >92% purity. Ethyl‑4‑(trimethylammoniumtrifluoromethanesulfonate)benzoate, the fluorobenzoic acid precursor (also reported as ‘FB precursor’), was purchased from ABX (Germany) and was supplied in >95% purity.

**GMP implementation of [^18^F]FB-A20FMDV2**

*Automated GMP-compliant radiosynthesis of [^18^F]FB-A20FMDV2*

The synthesis of [^18^F]FB-A20FMDV2 was carried out on a Modular-Lab™ system (Eckert and Ziegler, Germany) consisting of solenoid valve modules, multi-position valve modules, a stopcock module, a syringe valve module, two heated reaction modules fitted with pneumatic lifts and semi-preparative HPLC incorporating variable wavelength UV (Gilson, UK) and PIN diode radioactivity detection (Carroll-Ramsey, USA).

[^18^F]Fluoride was produced on a Siemens RDS-111 Eclipse cyclotron by the ^18^O(p,n)^18^F reaction using a fluoride target filled with oxygen-18 enriched water. The [^18^F]fluoride was transferred with a sweep of argon gas from the cyclotron target to the hot cell.

The [^18^F]fluoride in solution in oxygen-18 enriched water was trapped onto a SepPak® Light Accell™ Plus QMA Carbonate cartridge. Following trapping, the fluoride was released into the reactor using 0.8 mL of a solution consisting of 1 mL of acetonitrile, 0.1 mL of water, 30 mg of Kryptofix® and 6 mg of K_2_CO_3_. The contents of the reactor were evaporated a first time (90 °C, 5 min) and the evaporation process was subsequently repeated following the addition of a further 1 mL of anhydrous acetonitrile (90 °C, 5 min).

A solution of the precursor, ethyl 4-(trimethylammoniumtrifluoromethanesulfonate) benzoate (7-10 mg in 1 mL of anhydrous acetonitrile), was added to the reactor containing the dried [^18^F]fluoride. The reaction mixture was heated to 90 °C for 10 min and then cooled down to 50 °C, prior to the addition of a solution of tetrabutylammonium hydroxide in acetonitrile (40 µL TBAOH 40% in water v/v in 600 µL of acetonitrile). The reaction mixture was then heated to 120 °C for 3 min. The reactor was cooled down to 25 °C and trifluoroacetic acid (1 mL of 0.1% TFA in water) was added. The resulting reaction mixture was diluted with 20 mL of water and passed through an activated Classic C18 SepPak® cartridge. The SepPak® cartridge was washed with 1 mL of water and the cartridge and lines were dried with a stream of nitrogen applied for five minutes. [^18^F]Fluorobenzoic acid was eluted from the SepPak® cartridge using 1 mL of anhydrous DMF. The [^18^F]FBA in DMF was dried by passing it through a 4 Å molecular sieve cartridge and directed to a 3 mL fritted syringe containing the resin bound peptide A20FMDV2 (30-35 mg in 100 µL of DMF) and a magnetic stirrer bar. A solution containing HATU/DIPEA in DMF (9-12 mg of HATU in 50 µL of anhydrous DMF and 20 µL of a 20% v/v solution of DIPEA in anhydrous DMF) was added manually from outside the hot cell by means of a syringe and external delivery line to the fritted syringe containing the resin and [^18^F]FBA mixture . The coupling reaction was performed for 10 min at room temperature with stirring. The resin-bound peptide was separated from unreacted [^18^F]FBA by filtration and was washed sequentially with DMF (2-3 mL) and methanol (2-3 mL) and dried under a stream of helium. The crude [^18^F]FB‑A20FMDV2 was cleaved from the resin using 600 µL of TFA/TIPS/water solution (95:2.5:2.5; v/v/v). Following separation of the resin and the cleavage mixture by filtration, the mixture was diluted with water (3 mL) prior to semi-preparative RP‑HPLC purification.

The semi-preparative purification was carried out on an Agilent ZORBAX SB300 C8 column (9.4 x 250 mm, 5 µm). UV (210 nm) and radioactivity traces were monitored and recorded. The product was eluted with a gradient starting isocratic at 10% solvent B for 5 min, then increasing from 10% B to 30% B over 5 min and from 30% B to 40% B over 60 min at 4 mL/min (A: 0.04% TFA in water; B: 95:5 acetonitrile/water (v/v)). The product fraction corresponding to [^18^F]FB-A20FMDV2 (t_R_=26±2 min) was collected and diluted with water (20 mL). The resulting solution was loaded onto a tC18 light Sep-Pak® cartridge and washed with water (5 mL). Counter ion exchange was then achieved by careful transfer of a sodium acetate solution (5 mL, 100 mM) through the Sep‑Pak® cartridge to yield the [^18^F]FB‑A20FMDV2 acetate salt. [^18^F]FB-A20FMDV2 was eluted from the cartridge into a second reactor using a solution of 90% acetonitrile in water (450 µL). Reformulation of the product was achieved through evaporation of acetonitrile at 40 °C for 15 min under N_2_ flow. The dry residue was dissolved in 0.9% saline (3 mL) *in situ* and then transferred to a mixing vial containing a further 7 mL of 0.9% saline. Finally, the resulting [^18^F]FB-A20FMDV2 in 0.9% saline was filtered through a 0.2 µm sterile filter into its final sterile container.


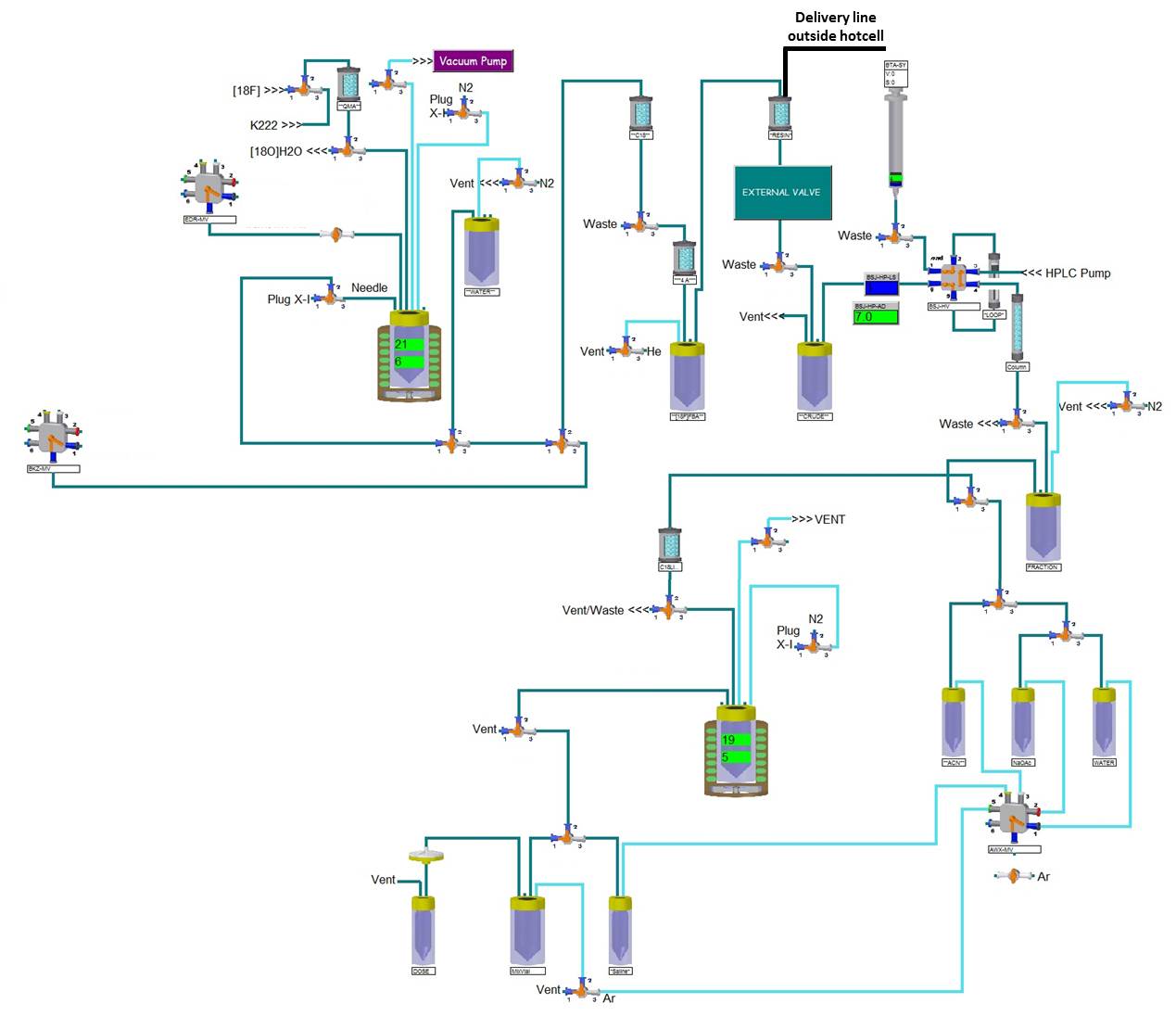


**Supplementary Fig 1** E&Z Modular-Lab scheme for the synthesis of [^18^F]FB-A20FMDV2

*Quality control of [^18^F]FB-A20FMDV2*

Final doses of [^18^F]FB-A20FMDV2 were tested using validated procedures in accordance with good manufacturing practices for: visual appearance, pH, total endotoxins, FB-A20FMDV2 mass, FB-A20FMDV2 precursor, total unknown chemical impurities, trifluoroacetic acid content, Kryptofix (K222) content,  radiochemical purity, radiochemical identity, isotopic half-life, filter integrity, isotopic purity, residual solvents content and sterility.

Quality control HPLC analysis was performed on an Agilent 1100 Series HPLC instrument consisting of a binary pump, degasser, column oven, autosampler, UV-vis/diode array detector and a sodium iodide detector (Berthold Technologies, Germany). Radioactivity measurement and radionuclidic identity were obtained using a calibrated ion chamber (ISOMED 2000, MED Nuklear-Medizintechnik, Germany). A Canberra 802 Scintillation Detector (Canberra Industries, UK) was used to determine the radionuclidic purity. Ion chromatography analysis was performed on a Dionex ICS-3000 system consisting of an auto-sampler, pump, column oven, anion suppressor and conductivity detector. pH measurement was performed on a Jenway Model 3510 pH/mV/temperature meter. Residual solvents were quantified by gas chromatography using an Agilent 6890 GC system equipped with a FID detector and an Agilent 123-1334 DB-624 capillary column (0.32 mm x 30 m). Endotoxin detection was performed by *Limulus amebocyte* lysate (LAL) test using a PTS100 Endosafe® Portable Test System (Charles River Laboratories, UK). Filter integrity was checked using a Millipore Integritest IT4N instrument (Millipore, UK).

The identity and purity (chemical and radiochemical) of [^18^F]FB-A20FMDV2 were determined by HPLC analysis using a Phenomenex Jupiter Proteo C12 column (4 μm, 90 Å; 4.6 x 250 mm) at 25 °C. The HPLC analysis method employed a gradient using acetonitrile and 0.04% TFA at 1.2 mL/min as described on Table 1.

**Supplementary Table 1** QC HPLC method for the analysis of [^18^F]FB-A20FMDV2

| **Time (min)** | **% Acetonitrile** | **% (0.4% TFA**  **in water)** |
| --- | --- | --- |
| 0.0 | 25 | 75 |
| 0.5 | 25 | 75 |
| 0.6 | 30 | 70 |
| 13.0 | 30 | 70 |
| 13.1 | 25 | 75 |
| 16.0 | 25 | 75 |

*Radiometabolite analysis of [^18^F]FB-A20FMDV2*

Blood samples used for radiometabolite analysis were centrifuged to obtain plasma (6080 x g, 3 min, 4 °C). The plasma was mixed with urea and the mixture diluted with 10 mM TRIS buffer (pH=8.0) in a 1:1 ratio. Samples were filtered through a 0.45 µm PVDF filter membrane prior to injection onto a trap and release HPLC method for analysis (Hilton, 2000).

Plasma samples were analysed using an Agilent 1200 series HPLC system using on-line extraction method. Sample was loaded onto the extraction column (OASIS HLB; 4.6 x 20 mm, 25 µm) using 5% acetonitrile in water as mobile phase (2.0 mL/min). At 4 minutes the valve was switched for loading the sample onto the analytical column (Agilent ZORBAX Eclipse XDB-C18; 4.6 x 150 mm, 5 µm). Mobile phase used for elution of radiometabolites consisted of 10 mM tris buffer (pH=8.0) and acetonitrile delivered at a flow rate of 2.0 mL/min in a 13-min gradient. The gradient profile is shown in Table 2. Representative chromatograms are presented in Fig 7.

**Supplementary Table 2** HPLC gradient profile for the radiometabolite analysis of [^18^F]FB-A20FMDV2

| **Time (min)** | **% Acetonitrile** |
| --- | --- |
| 0.00 | 5.0 |
| 4.00 | 5.0 |
| 8.00 | 40.0 |
| 10.00 | 60.0 |
| 12.00 | 95.0 |
| 13.00 | 95.0 |

***In vitro* selectivity of A20FMDV2**

A20FMDV2 competition binding studies against α_v_β_1_, α_v_β_3_, α_v_β_5_, α_v_β_6_ and α_v_β_8_ were completed using radioligand binding assays, as previously described [1]. The same assay format was also used for competition binding against α_5_β_1_ (1.5 nM protein) and α_8_β_1_ (0.75 nM protein) using an incubation time of 6 h. To determine the binding of A20FMDV2 to the α_IIb_β_3_ integrin, inhibition of fibrinogen-induced platelet aggregation was measured as previously described [2]. Total and non-specific binding values were measured in the presence of vehicle (0.1 % DMSO for α_IIb_β_3_ and 1% DMSO for all remaining RGD integrins) or 3 µM Tirofiban (α_IIb_β_3_ only)/10 µM SC-68448 (for all remaining RGD integrins) respectively, and were used to calculate the % inhibition of RGD ligand bound to each integrin. Where applicable data were fitted using non-linear regression analysis (four-parameter logistic equation with variable slope) and pIC50 values generated were converted to pKI values using the Cheng-Prusoff equation [3].

**Preclinical rodent studies**

Animals

Male Sprague-Dawley rats (Charles River, Kent, UK), housed under a 12-h light/dark cycle (07:00h lights on) with food and water available *ad libitum* were allowed to habituate for at least 7 days before performing experimental procedures.

PET-CT Imaging

All PET-CT imaging was carried out using a Siemens Inveon DPET/MM PET-CT scanner (Siemens AG, Erlangen, Germany). A 15-min CT scan was performed to collect data for attenuation and scatter correction and to provide structural information. Subsequently, dynamic PET images were acquired, which were divided into pre-defined frames. 3D histograms with span 3 and maximum ring difference of 79 were used. Fourier rebinning was performed and images reconstructed using a 2D FBP algorithm and a ramp filter to generate images on a 128x128 matrix.

Body temperature and respiratory rate were monitored using a rectal probe and respiration pad, respectively. Body temperature was maintained using a heating mat and lamp.

Image processing and data analysis were performed using an in-house semi-automatic computational pipeline derived from MIAKAT™ ([www.miakat.org](http://www.miakat.org)). The images generated by the Inveon scanner were converted into Nifti format and were quality checked for any misalignment between PET and CT. The dynamic PET images were manually re-aligned to the CT images if any misalignment was observed. ROIs were manually defined in itkSNAP ([www.itksnap.org](http://www.itksnap.org)) using both anatomical information (CT images) and summed PET images comprising early (0–5 min) and late (10–60 min) stages of the radiotracer uptake. The ROIs were applied to the dynamic PET data to generate the corresponding time–activity curves (TACs) for each region of interest. Tissue radioactivity concentrations were normalized for the radioactive dose injected and for the animal's body mass giving standard uptake values ((kBq.ml^−1^ tissue)/(kBq.g^−1^ body mass)) with the assumption that 1 cm^3^ equals 1 g of tissue.

Image analysis of *in vivo* competition studies

Time activity curves (TACs) for two ROIs (lung and heart chamber) were generated. Semi-quantitative parameters were derived from the TACs:

SUVR_30-60_: the ratio between the SUV_30-60_ in the ROI (lung) and the reference region (heart chamber) reflecting the blood concentration of the radiotracer.

The specific component of the signal was assessed by calculating the reduction of this signal post-cold radiotracer (homologous competition) or post antibody (heterologous competition) using the following equation;

$$\Delta\mathrm{SUVR}_{30-60}\left( \% \right)=100\times\frac{{\mathrm{SUVR}_{30-60}}^{\mathrm{postdose}}-{\mathrm{SUVR}_{30-60}}^{\mathrm{baseline}}}{{\mathrm{SUVR}_{30-60}}^{\mathrm{baseline}}}.$$

*In-vivo homologous competition study*

*
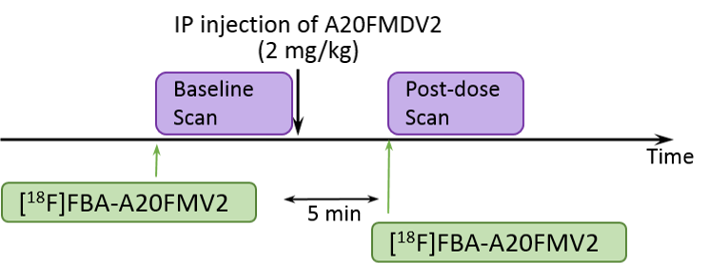
*

**Supplementary Fig. 2** Schematic of the study design for the homologous blocking study

*In-vivo heterologous competition study*


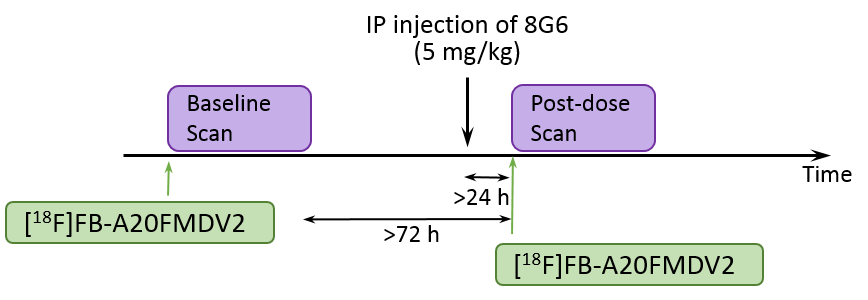


**Supplementary Fig. 3** Schematic of the study design for the heterologous blocking study. 8G6 at 5 mg/kg engages α_v_β_6_ pharmacology [4, 5]

Assay method for 8G6 antibody content

Rat blood:water (50:50 v/v) samples were analysed for 8G6 mouse anti-α_V_β_6_ monoclonal antibody using a validated analytical method on the Gyrolab platform. Total antibody levels were determined using a generic assay using biotinylated anti mouse IgG to capture mouse anti-α_V_β_6_ and an Alexa labelled 647 labelled anti-rat/mouse IgG was used as the detection antibody. The lower limit of quantification (LLQ) was 300 ng/mL and the higher limit of quantification (HLQ) was 30,000 ng/mL using a 3 µL aliquot of rat blood:water (50:50) diluted 1/50 with Rexxip A buffer.

Quality Control samples (QC), prepared at three different analyte concentrations and stored with study samples, were analysed with each batch of samples against separately prepared calibration standards. For the analysis to be acceptable, at least 3 out of 6 QC results should not deviate from the nominal concentration by more than 20%, and at least 50% of the results from each QC concentration should be valid (<20%). The applicable analytical runs met all predefined run acceptance.

*Rodent dosimetry*

The following tissues were collected: bladder content, bladder wall, pancreas, stomach wall, stomach content, small intestine wall, small intestine content, upper large intestine wall, upper large intestine content, colon wall, colon content, red bone marrow, bone surface, muscle, spleen, liver, testes, adrenals, kidney, lung, heart, oesophagus, skin, thymus, thyroid, brain and salivary gland. Blood, plasma and tissue radioactive counts were measured in a multi-well gamma counter (1470 Wizard, Perkin Elmer, Waltham, MA, USA), decay corrected to the incubation time point for each rat and expressed as the percentage dose per gram of wet tissue (%ID/g).

The mean values obtained from all rats were integrated over time to derive residence times for the organs of interest. The expected human dosimetry for [^18^F]FB-A20FMDV2 was calculated using whole-body distribution data from the rat, scaled to reflect human values based upon the relative total body mass of rat and human, and the mass of each human organ. The activity not accounted for was assumed to be evenly distributed, represented by a ‘remaining body’ residence time. The determined residence times were entered into the OLINDA/EXM v1.1 software (Organ Level Internal Dose Assessment Code, Vanderbilt University, Nashville, TN, USA) to estimate the organ absorbed doses per unit of administered activity and derive the total effective dose using the ‘adult male’ hermaphrodite human dosimetry phantom. The organs used in OLINDA [6] as both sources and targets follow the ICRP60 standard (ICRP 1990).

**Results**

**GMP implementation of [^18^F]FB-A20FMDV2**

*Quality control of [^18^F]FB-A20FMDV2*

Supplementary Fig. 4 Example of [^18^F]FB-A20FMDV2 analytical HPLC chromatograms (sample). (Top) Radioactive trace. (Bottom) UV trace (210 nm)

Supplementary Fig. 5 Example of [^18^F]FB-A20FMDV2 analytical HPLC chromatograms (spiked sample). (Top) Radioactive trace. (Bottom) UV trace (210 nm)

**Supplementary Table 3** [^18^F]FB-A20FMDV2 quality control tests and specifications

| **Quality Aspect** | **Quality Specification**  **(in total administered dose)** | **Method** |
| --- | --- | --- |
| Radiochemical identity | Concordant with FB-A20FMDV2 reference standard | HPLC  (radioactivity & UV detection) |
| Radiochemical purity | ≥ 95% | HPLC (radioactivity detection) |
| Radioactivity content | 50-500 MBq | Dose calibrator |
| Appearance | Clear, colourless, free from particles | Visual |
| Radioactive half-life | 109.7 min ± 5% | Calculation from dose calibrator |
| Gamma energy | Concordant with F-18 energy spectrum | Gamma spectrometer |
| Impurities (chemical): |  | HPLC |
| A20FMDV2 precursor | Not more than 10 µg | UV detection |
| Total unknown impurities | Not more than 12 µg |  |
| Kryptofix® 2.2.2 | Not more than 2.2 mg | Thin layer chromatography |
| FB-A20FMDV2 | Not more than 12.5 µg | Radio-HPLC |
| pH | 4.5 – 8.5 | pH meter |
| Residual solvents:  Acetonitrile  DMF  Methanol | (in total administered dose)  Not more than 4.1 mg  Not more than 8.8 mg  Not more than 30 mg | Gas chromatography |
| Amount of residual TFA: | ≤ 2.0 ppm | Ion exchange chromatography |
| Bacterial endotoxins | Not more than 175 EU Total  (endotoxins of gram negative bacterial origin) | Charles River Endosafe®-PTS™  (according to Ph.Eur. 2.6.14) |
| Sterility | Complies with Ph. Eur.  (membrane filtration sterility test) | Sterility test (according to Ph.Eur. 2.6.1) |
| Filter integrity of product filter | Filter is integral | Millipore Integritest® 4 Filter integrity test |
| Filter integrity of vent filter | Filter is integral | Millipore Integritest® 4 Filter integrity test |

**Supplementary Table 4** Data for the three validation batches of [^18^F]FB-A20FMDV2


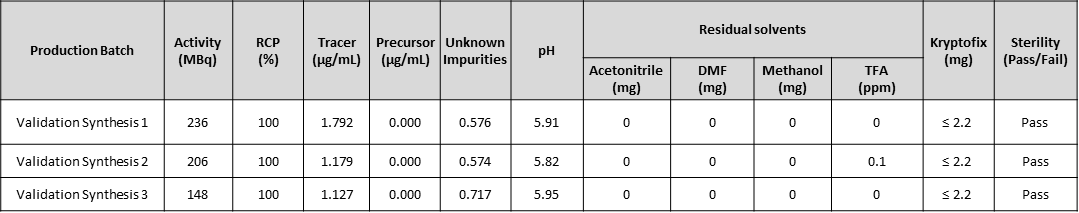


**Preclinical rodent studies**

*In vivo homologous competition study*

**Supplementary Table 5** Molar activity, injected radioactivity and injected mass of [^18^F]FB-A20FMDV2

| **Homologous**  **competition study** | | **Molar activity**  **GBq/µmol** | **Injected radioactivity**  **(MBq)** | **Mass injected (µg)** |
| --- | --- | --- | --- | --- |
| **Rat 1** | **Baseline scan** | 36.5 | 19.19 | 1.20 |
|  | **Post-dose scan** | 3.16 | 17.59 | 12.73 |
| **Rat 2** | **Baseline scan** | 39.6 | 15.63 | 0.90 |
|  | **Post-dose scan** | 0.98 | 15.16 | 35.29 |
| **Rat 3** | **Baseline scan** | 8.14 | 7.88 | 2.21 |
|  | **Post-dose scan** | 0.61 | 4.90 | 18.35 |

*In vivo heterologous competition study*

**Supplementary Table 6** Molar activity, injected radioactivity and injected mass of [^18^F]FB‑A20FMDV2

| **Heterologous**  **competition study** | | **Molar activity**  **(GBq/µmol)** | **Injected radioactivity**  **(MBq)** | **Mass injected (µg)** |
| --- | --- | --- | --- | --- |
| **Rat 1** | **Baseline scan** | 14.5 | 5.7 | 0.899 |
|  | **Post-dose scan** | 4.48 | 1.27 | 0.65 |
| **Rat 2** | **Baseline scan** | 3.58 | 10.9 | 6.93 |
|  | **Post-dose scan** | 63.3 | 10.7 | 0.387 |
| **Rat 3** | **Baseline scan** | 1.39 | 10.8 | 17.7 |
|  | **Post-dose scan** | 30.1 | 10 | 0.759 |
| **Rat 4** | **Baseline scan** | 11.34 | 10.4 | 2.08 |
|  | **Post-dose scan** | 11.39 | 7.32 | 1.47 |
| **Rat 5** | **Baseline scan** | 6.41 | 8.19 | 2.92 |
|  | **Post-dose scan** | 6.56 | 6.94 | 2.42 |
| **Rat 6** | **Baseline scan** | 14.92 | 2.62 | 0.401 |
|  | **Post-dose scan** | 27.72 | 6.64 | 0.547 |

The mass of unlabelled FB-A20FMDV2 injected does not show any correlation with the SUV observed.


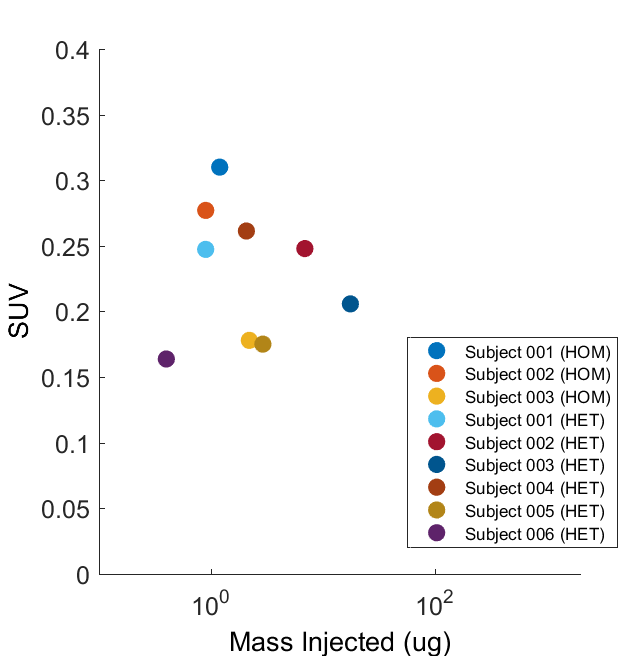


Supplementary Fig. 6 SUV related to the mass of unlabelled FB-A20FMDV2 injected at each baseline scanning session. Each colour represents an animal

*Rodent radiodosimetry*

Rodent biodistribution was utilised to estimate human radiation exposure using OLINDA/EXM [6]. The highest activity concentration was observed in urine, followed by the small intestine (wall and content), the kidney and liver.

Dosimetry calculations provide the individual organ doses and the whole body effective dose. The organ absorbed doses estimated using OLINDA/EXM software are summarised in **Table 7**. Data revealed the organ with the highest absorbed dose and contribution to the effective dose was the bladder. The resultant effective dose in humans was estimated to be 0.0335 mSv/MBq.

**Supplementary Table 7** Organ effective doses (ED, mSv/MBq injected) for the ‘adult male’ human hermaphrodite phantom derived by OLINDA/EXM

| **Target organ** | **Absorbed dose (mGy/MBq)** | | | | | | | | | | **Effective Dose Contribution (mSv/MBq)** |
| --- | --- | --- | --- | --- | --- | --- | --- | --- | --- | --- | --- |
|  | | **Beta** | **Gamma** | | | **Total** | | | | |  |
| Adrenals | 2.42E-03 | | | 7.46E-03 | | | 9.89E-03 | | 4.94E-05 | | |
| Brain | 4.91E-05 | | | 1.67E-03 | | | 1.72E-03 | | 8.59E-06 | | |
| Breasts | 2.42E-03 | | | 4.13E-03 | | | 6.55E-03 | | 3.28E-04 | | |
| Gallbladder wall | 2.42E-03 | | | 1.11E-02 | | | 1.35E-02 | | 0.00E+00 | | |
| Lower large intestine wall | 3.15E-03 | | | 2.03E-02 | | | 2.34E-02 | | 2.81E-03 | | |
| Small intestines | 4.27E-02 | | | 1.90E-02 | | | 6.17E-02 | | 3.08E-04 | | |
| Stomach wall | 6.26E-03 | | | 1.01E-02 | | | 1.64E-02 | | 1.96E-03 | | |
| Upper large intestine | 2.81E-03 | | | 1.78E-02 | | | 2.06E-02 | | 1.03E-04 | | |
| Heart wall | 9.65E-04 | | | 4.54E-03 | | | 5.50E-03 | | 0.00E+00 | | |
| Kidneys | 2.70E-02 | | | 1.44E-02 | | | 4.14E-02 | | 2.07E-04 | | |
| Liver | 6.73E-03 | | | 8.87E-03 | | | 1.56E-02 | | 7.80E-04 | | |
| Lungs | 1.60E-03 | | | 4.30E-03 | | | 5.91E-03 | | 7.09E-04 | | |
| Muscle | 5.22E-04 | | | 7.77E-03 | | | 8.29E-03 | | 4.15E-05 | | |
| Ovaries | 2.42E-03 | | | 2.15E-02 | | | 2.40E-02 | | 4.79E-03 | | |
| Pancreas | 4.42E-04 | | | 7.24E-03 | | | 7.68E-03 | | 3.84E-05 | | |
| Red marrow | 1.91E-03 | | | 7.65E-03 | | | 9.57E-03 | | 1.15E-03 | | |
| Osteogenic cells | 6.55E-03 | | | 6.69E-03 | | | 1.32E-02 | | 1.32E-04 | | |
| Skin | 2.42E-03 | | | 4.19E-03 | | | 6.61E-03 | | 6.61E-05 | | |
| Spleen | 3.04E-04 | | | 5.82E-03 | | | 6.12E-03 | | 3.06E-05 | | |
| Testes | 3.55E-04 | | | 1.13E-02 | | | 1.16E-02 | | 0.00E+00 | | |
| Thymus | 2.42E-03 | | | 4.69E-03 | | | 7.11E-03 | | 3.56E-05 | | |
| Thyroid | 6.70E-04 | | | 3.99E-03 | | | 4.66E-03 | | 2.33E-04 | | |
| Urinary bladder wall | 2.69E-01 | | | 1.21E-01 | | | 3.90E-01 | | 1.95E-02 | | |
| Uterus | 2.42E-03 | | | 3.50E-02 | | | 3.74E-02 | | 1.87E-04 | | |
| Total body | 3.53E-03 | | | 7.51E-03 | | | 1.10E-02 | | 0.00E+00 | | |
| **Effective dose (mSv/MBq)** | | | | |  | | |  | | **3.35E-02** | |


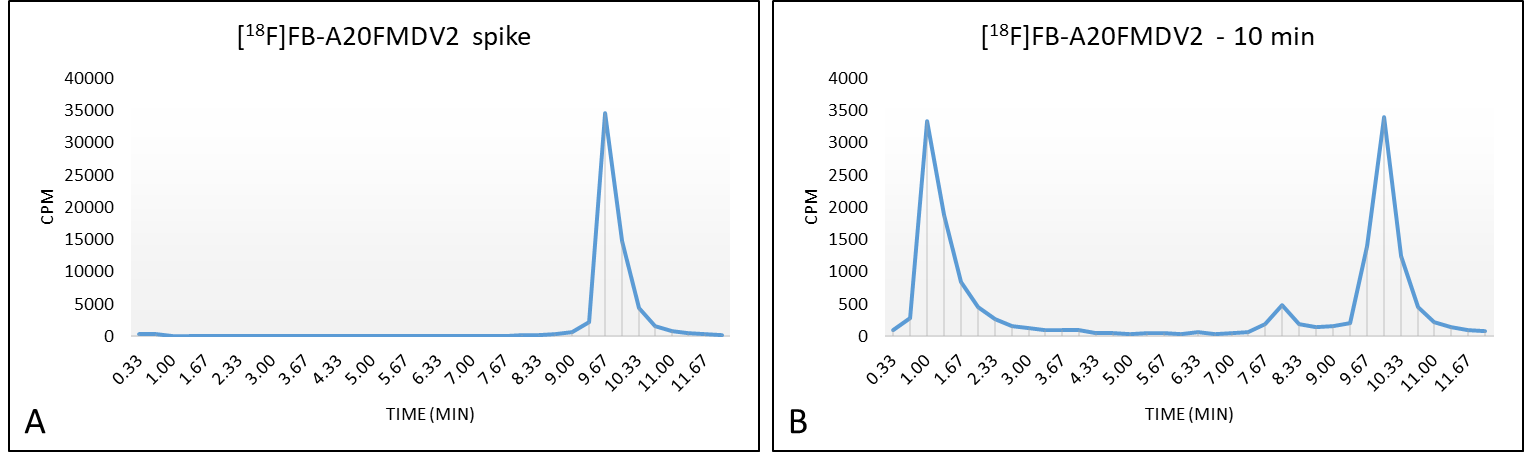


Supplementary Fig. 7 A) Representative radiochromatogram of authentic [^18^F]FB-A20FMDV2 and B) representative chromatogram of [^18^F]FB-A20FMDV2 10min post iv administration.

**References**

1. Rowedder JE, Ludbrook SB, Slack RJ. Determining the true selectivity profile of αv integrin ligands using radioligand binding: Applying an old solution to a new problem. SLAS Discovery 2017;22:962-973.
2. Hall ER, Bibby LI, Slack RJ. Characterisation of a novel, high affinity and selective αvβ6 integrin RGD-mimetic radioligand. Biochem Pharmacol. 2016;117:88-96.
3. Cheng Y, Prusoff WH. Relationship between the inhibition constant (K1) and the concentration of inhibitor which causes 50 per cent inhibition (I50) of an enzymatic reaction. Biochem Pharmacol. 1973;22:3099–3108.
4. Horan GS, Wood S, Ona V, et al. Partial inhibition of integrin alpha(v)beta6 prevents pulmonary fibrosis without exacerbating inflammation. Am J Respir Cri Care Med. 2008;177:56–65.
5. Hahm K, Lukashev ME, Luo Y et al. Alphav beta6 integrin regulates renal fibrosis and inflammation in Alport mouse. Am J Pathol. 2007;170:110–125.
6. Stabin MG, Sparks RB, Crowe E. OLINDA/EXM: the second-generation personal computer software for internal dose assessment in nuclear medicine. J Nucl Med. 2005;46:1023–1027.
